# *Klebsiella quasipneumoniae* in intestine damages bile acid metabolism in hematopoietic stem cell transplantation patients with bloodstream infection

**DOI:** 10.1186/s12967-023-04068-9

**Published:** 2023-03-29

**Authors:** Guankun Yin, Yifan Guo, Qi Ding, Shuai Ma, Fengning Chen, Qi Wang, Hongbin Chen, Hui Wang

**Affiliations:** 1 Department of Clinical Laboratory, Peking University People’s Hospital, Beijing, 100044 China; 2 Institute of Medical Technology, Peking University Health Science Center, Beijing, 100191 China

**Keywords:** Hematopoietic stem cell transplantation, Bloodstream infection, Intestinal microbiome, *Klebsiella quasipneumonae*, Serum metabolome, Primary bile acid

## Abstract

**Background:**

Bloodstream infection (BSI) is a serious hematopoietic stem cell transplantation (HSCT) complication. The intestinal microbiome regulates host metabolism and maintains intestinal homeostasis. Thus, the impact of microbiome on HSCT patients with BSI is essential.

**Methods:**

Stool and serum specimens of HSCT patients were prospectively collected from the pretransplant conditioning period till 4 months after transplantation. Specimens of 16 patients without BSI and 21 patients before BSI onset were screened for omics study using 16S rRNA gene sequencing and untargeted metabolomics. The predictive infection model was constructed using LASSO and the logistic regression algorithm. The correlation and influence of microbiome and metabolism were examined in mouse and Caco-2 cell monolayer models.

**Results:**

The microbial diversity and abundance of Lactobacillaceae were remarkably reduced, but the abundance of Enterobacteriaceae (especially *Klebsiella quasipneumoniae*) was significantly increased in the BSI group before onset, compared with the non-BSI group. The family score of microbiome features (Enterobacteriaceae and Butyricicoccaceae) could highly predict BSI (AUC = 0.879). The serum metabolomic analysis showed that 16 differential metabolites were mainly enriched in the primary bile acid biosynthesis pathway, and the level of chenodeoxycholic acid (CDCA) was positively correlated with the abundance of *K. quasipneumoniae* (*R* = 0.406, *P* = 0.006). The results of mouse experiments confirmed that three serum primary bile acids levels (cholic acid, isoCDCA and ursocholic acid), the mRNA expression levels of bile acid farnesol X receptor gene and apical sodium-dependent bile acid transporter gene in *K. quasipneumoniae* colonized mice were significantly higher than those in non-colonized mice. The intestinal villus height, crypt depth, and the mRNA expression level of tight junction protein claudin-1 gene in *K. quasipneumoniae* intestinal colonized mice were significantly lower than those in non-colonized mice. In vitro, *K. quasipneumoniae* increased the clearance of FITC-dextran by Caco-2 cell monolayer.

**Conclusions:**

This study demonstrated that the intestinal opportunistic pathogen, *K. quasipneumoniae*, was increased in HSCT patients before BSI onset, causing increased serum primary bile acids. The colonization of *K. quasipneumoniae* in mice intestines could lead to mucosal integrity damage. The intestinal microbiome features of HSCT patients were highly predictive of BSI and could be further used as potential biomarkers.

**Supplementary Information:**

The online version contains supplementary material available at 10.1186/s12967-023-04068-9.

## Background

Hematopoietic stem cell transplantation (HSCT) is used to treat various benign and malignant hematologic diseases. Complications, including graft-versus-host disease (GVHD) and infection, are common and life-threatening. Bloodstream infection (BSI) is closely associated with high morbidity and mortality in HSCT patients [1, 2]. Bacterial or fungal infections after HSCT are found in many patients [3]. Therefore, early BSI detection is of great significance for clinical treatment. Recently, the connection between the microbiome and microbiome-related metabolites and HSCT complications has been increasing [4]. The characteristics and relationship between the intestinal microbiome and host metabolome before BSI remain to be investigated.

The intestinal microbiome is crucial for maintaining and promoting human health, maintaining intestinal immune homeostasis, resisting exogenous microorganism invasion, and protecting intestine from damage [5]. However, intestinal microbiome destruction is also associated with various diseases [6–11]. Recently, microbiome characterisation has made it possible to better understand the complex interactions between the microbiome and HSCT [4]. Studies have shown that obligate anaerobic symbiotic bacteria in the intestine play a key role in maintaining the normal intestinal environment balance [3]. HSCT patients usually manifest with the loss of obligate anaerobic symbionts, pathogen expansion, and overall microbial diversity reduction [4]. Studies have reported that intestinal microbiome is related to infection; for example, *Enterococcus* or Proteobacteria increase bacteraemia risk [12, 13]. Gram-negative dominance in the intestine is also associated with BSI [14]. Furthermore, a single-centre observational study found that Gammaproteobacteria is a predictor of pulmonary complications after HSCT [15, 16].

Changes in host metabolites caused by alterations in the intestinal microbiome’s structure and density were associated with GVHD occurrence [17, 18]. Butyrate is related to intestinal microbiome diversity in patients with GVHD [19, 20]. A high abundance of butyrate-producing bacteria reduces the risk of lower respiratory tract virus infection in patients after allo-HSCT [10]. Bile acids and plasmalogens vary at acute GVHD onset [18]. However, there has been little evidence of metabolic features in HSCT patients with BSI. This study aimed to investigate the biological characteristics preceding BSI onset by analysing intestinal microbiome and serum metabolome, and further determine their potential associations with BSI onset.

## Methods

### Study design, patient and specimen collection

**Fig. 1:**
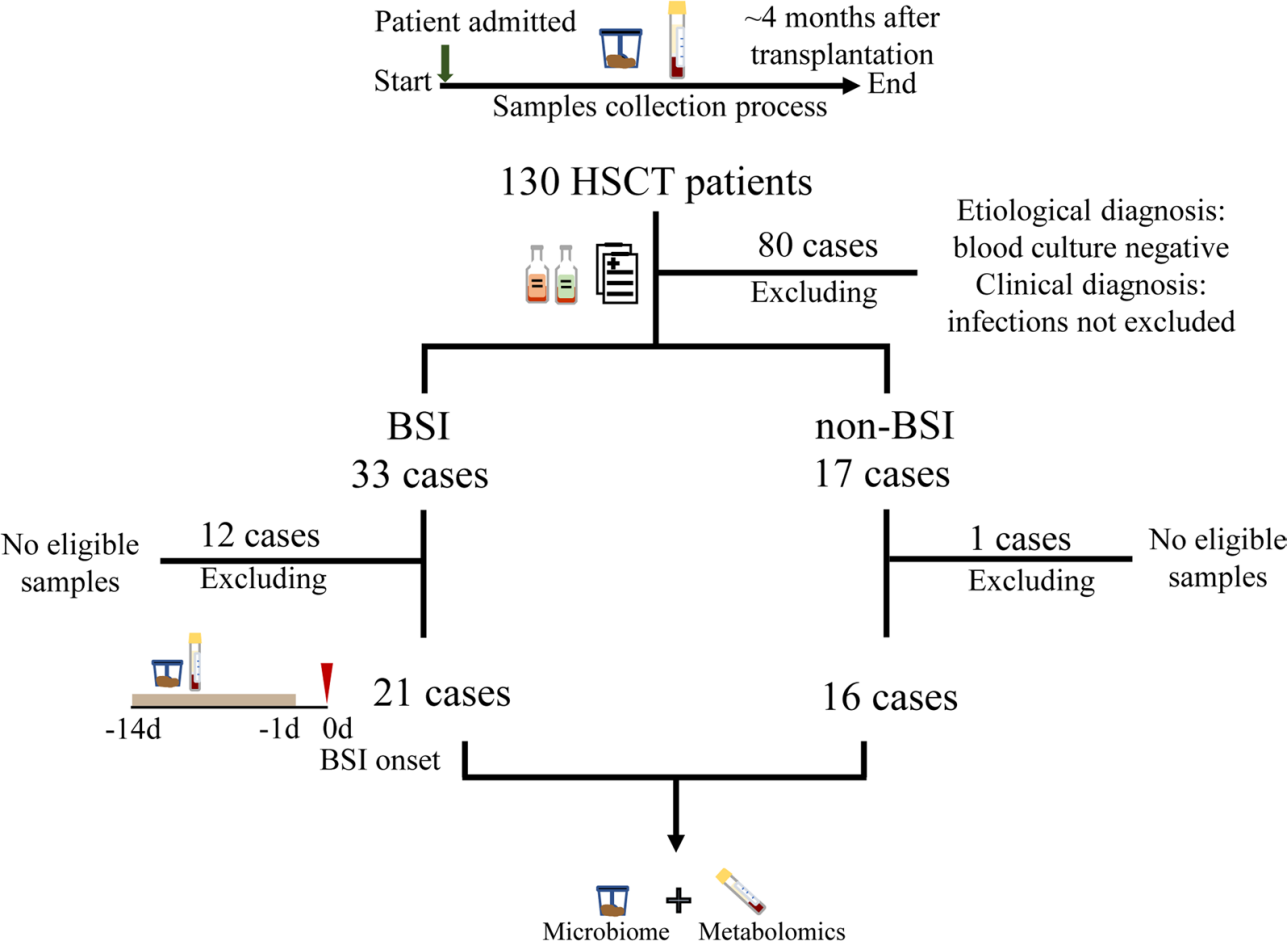
Experimental design process. The experimental design process included the sample collection process, patient screening and sample detection. Brown bar represented that the samples were within 14 days before BSI onset

A total of 130 HSCT patients at Peking University People's Hospital from June 2020 to February 2021 were enrolled. Specimens (stool and serum) from each patient were prospectively collected from the pretransplant conditioning period to 4 months after transplantation. According to the BSI diagnostic criteria [21], 33 BSI and 17 non-BSI patients were screened. The remaining 80 patients were excluded because their blood culture results were negative, but infections were not ruled out clinically (Fig. 1). Finally, 21 BSI patients with paired specimens (stool and serum) within 30 days after transplantation and within 14 days before BSI onset were included, and 16 non-BSI patients with paired specimens within 30 days after transplantation were screened (Fig. 1 and Additional file 2: Table S1). The specimens of BSI and non-BSI group were 29 and 16 pairs, respectively.

BSI diagnostic criteria were based on laboratory-confirmed bloodstream infection criteria from the Centers for Disease Control and Prevention’s National Healthcare Safety Network, and BSI onset was defined as the time of collecting the first positive blood culture [21]. The study was approved by the ethics committees of Peking University People's Hospital (No. 2021PHB414-002) and all remaining clinical samples were obtained consent from patients.

### 16S rRNA gene sequencing in human stool and data analysis

All clinical residual stool specimens were collected and refrigerated at − 80 ℃ until tested. Genomic DNA of the stool specimens was extracted using the DNeasy PowerSoil Pro Kit (QIAGEN, Germany). The extracted DNA from each specimen was used as a template to amplify the V3–V4 regions of 16S rRNA genes using PCR. The PCR products were detected using agarose gel electrophoresis, and the target products were purified using a Gel Extraction Kit (QIAGEN, Germany). The library was constructed using a TruSeq® DNA PCR-Free Sample Preparation Kit (Illumina, USA). The purified products were sequenced using the Illumina NovaSeq6000 platform (Illumina, USA), and 250 bp paired-end reads were generated.

The barcodes and primer sequences were removed from the raw data. Paired-end reads were assembled using FLASH (version 1.2.7) [22]. QIIME (version 1.9.1) [23] was used to filter high-quality clean tags, and chimeric sequences were removed. Operational taxonomic units (OTU) clustering was performed using UPARSE software (version 7.0.1001) [24]. Species annotation was performed using the Silva database based on the Mothur algorithm [25]. OTUs abundance information were normalized using a standard of sequence number corresponding to the sample with the least sequences. The subsequent alpha and beta diversity analyses were based on the normalized data (see Additional file 1: Supplementary Methods for further details).

### Construction of infection prediction model

The following steps were completed for the family score calculation using the R statistical software (version 4.2.0). The “createDataPartition” package was used to divide the BSI and non-BSI groups into the training and validation sets randomly (training set: validation set = 7:3) to perform Least absolute shrinkage and selection operator (LASSO) algorithm (Additional file 7: Fig. S2). The “glmnet” package was used to perform the LASSO algorithm as previously described [26]. Coefficients reduced to zero were excluded. The remaining coefficients were analysed using logistic regression. The "glm" and "predict" functions were used to perform logistic regression and calculate the prediction value called "family score" in this study, respectively. The "boot" package was used to complete the bootstrap method to validate the regression model (Sampling with replacement). The number of bootstrap replicates was set at 1000. The coefficients after bootstrapping were listed in Additional file 3: Table S2.

### Untargeted metabolomic analysis in human serum

The remaining clinical serum specimens were refrigerated at − 80 °C until tested. Ice-cold methanol was added to the serum. The mixture was then incubated and centrifuged. The supernatant was collected for LC–MS/MS analysis. LC–MS/MS analysis was performed using a QTOF/MS-6545 (Agilent, USA) and 1290 Infinity LC (Agilent, USA). The HPLC conditions were as follows: UPLC: column, ACQUITY UPLC HSS T3 C18, 1.8 µm, 2.1 mm × 100 mm (Waters, USA); column temperature, 40 ℃; flow rate, 0.4 mL/min; injection volume, 2 μL; solvent system, water (0.1% formic acid) and acetonitrile (0.1% formic acid). Information on the specimens was acquired using the LC–MS system, followed by machine orders. The original data were transformed into the mzML format using ProteoWizard software (version 3.0). Peak extraction, alignment, and retention time correction were performed using the XCMS program [27]. Metabolic identification information was obtained by searching the Pubchem database, KEGG database, the Human Metabolome database [27]. The differential metabolites were filtered according to *P* value < 0.05, |log_2_FC| > 1, and VIP ≥ 1 (see Additional file 1: Supplementary Methods for details).

### Mice

Female C57BL/6N mice (4–8 weeks old) were purchased from Beijing Vital River Laboratory Animal Technology Co., Ltd (Beijing, China). All experimental mice were no more than five in a cage. Twelve-hour light–dark cycles were carried out at 20–22 ℃. The feed and bedding were sterile. The Peking University People's Hospital Animal Care Committee approved all procedures.

### Intestinal microbiome depletion, chemotherapy and bacterial colonization in mice

The mice were administered broad-spectrum antibiotics (metronidazole 1 g/L, neomycin sulfate 1 g/L, ampicillin 1 g/L and vancomycin 0.5 g/L (MNVA)) in drinking water for 14 days to deplete the microbiome, as described previously [28]. Chemotherapy was improved according to the previous method by continuous intraperitoneal injection of cytarabine 120 mg kg^−1^ d^−1^ and cyclophosphamide 100 mg kg^−1^ d^−1^ for 4 days [29]. The mice were administered suspension of *K. quasipneumoniae* or supernatant of stool suspension by gavage for 3 days. The amount of *K. quasipneumoniae* was 8 × 10^8^ CFU per mouse per day. Five (5/21) BSI and 3 (3/16) non-BSI patients’ stool specimens were randomly selected to prepare the stool supernatant, respectively. Each patient’s stool (0.5 g) was placed in 1 × PBS and resuspended. The stool suspension was filtered with cell strainers and then centrifuged (600×*g*, 5 min). The supernatant was separated and added to 50% glycerol to freeze-store until transplantation.

### 16S rRNA gene copy numbers detection of mice stools

The mice stool samples before and after administering broad-spectrum antibiotics were collected and kept at − 80 ℃. DNA from 20 to 25 mg of mouse stools was extracted using a DNeasy PowerSoil Pro Kit (QIAGEN, Germany). DNA concentrations were measured using a Qubit dsDNA HS Assay Kit (Thermo Fisher Scientific, USA). The 16S rRNA gene was quantified using real-time PCR, and the copy numbers were calculated [30]. The 16S rRNA gene primer sequences were used as previously described [30]. Forward primer: (5′ to 3′) TCCTACGGGAGGCAGCAGT; reverse primer: (5′ to 3′) GGACTACCAGGGTATCTAATCCTGTT. Real-time PCR was performed using the ABI Prism 7500 system (Applied Biosystems, USA) with TB Green® Premix Ex Taq™ II (Takara, China).

### Quantitative real-time reverse transcription-PCR of mice small intestine

Total RNA in mice small intestine was extracted using Quick-RNA Miniprep Plus Kit (ZYMO Research, USA) and synthesized cDNA using PrimeScript RT Master Mix (Takara, China). The primers used were shown in Additional file 6: Table S5 and *β*-actin was used as internal reference gene. Real-time PCR was performed using the ABI Prism 7500 system (Applied Biosystems, USA) with TB Green® Premix Ex Taq™ II (Takara, China).

### Bile acid detection in mice serum

Mouse serum (50 μL) was added with 200 μL methanol/acetonitrile. Ten microlitres of an internal standard mixed solution (1 μg/mL) were added to the extract as an internal standard for quantification. Samples were taken at − 20 °C for 10 min. After centrifuging for 10 min (12,000 r/min, 4 °C), the supernatant was evaporated to dryness and reconstituted in 100 μL of 50% methanol for further LC–MS/MS analysis. The analysis was performed using an LC–ESI–MS/MS system (UHPLC, ExionLC™ AD; MS, Applied Biosystems 6500 Triple Quadrupole).

### Histology of small intestine in mice

The ileum (approximately 0.5 cm) was collected, washed with 1 × PBS, and placed in 4% paraformaldehyde. Intestinal samples in 4% paraformaldehyde were dehydrated and embedded in paraffin wax. Paraffin-embedded tissues were sectioned (approximate thickness 5 μm) and stained with haematoxylin and eosin (H&E). Three parts of each sample were randomly selected to measure villus height and crypt depth. Each part included at least three villi and crypts. For the goblet cells, five random vision fields at 40× magnification were determined to calculate the cell number, and each field contained three villi.

### Cell culture and fluorescein isothiocyanate (FITC)-dextran transport

Caco-2 cells were cultured in Dulbecco's Modified Eagle Medium/Nutrient Mixture F-12 medium (DMEM/F-12) supplemented with 20% (v/v) fetal bovine serum and non-essential amino acids at 37 ℃ and 5% CO_2_. Caco-2 cells were transferred on transwell inserts (pore size = 0.4 μm; Thermo Scientific™ Nunc™, Denmark) in 6-well plates (5 × 10^5^ cells/transwell) and cultured for 21 days to construct monolayers. The transepithelial electrical resistance (TEER) of monolayers were measured by Epithelial Volt-Ohm Meter (Millicell ERS-2, USA). The monolayers were incubated with 1 × 10^9^ CFU/mL *K. quasipneumoniae* or *Enterococcus faecium* for 1 h and with 100 mM chenodeoxycholic acid (CDCA; Sigma, Germany) for 24 h [31, 32]. After incubations, the paracellular transport was measured by the clearance of FITC-dextran (4 kDa, 100 μg/mL) (Sigma, Germany) [31]. The fluorescence was measured every hour for 4 h (λexc: 493 nm; λem: 520 nm).

### Statistics

Statistical analysis of all data was performed using R statistical software (version 4.2.0) and GraphPad Prism (version 8.0). The Shapiro–Wilk test was used to test normal distribution, and Fisher's exact test was used to test homogeneous variance. For data with normal distribution and homogeneous variance, one-way ANOVA was used to compare multiple data groups; an unpaired two-tailed Student's t-test was used to compare two data groups. For data with abnormal distribution or uneven variance, the Kruskal–Wallis test was used to compare multiple data groups; the Mann–Whitney *U* test was used to compare the two data groups. *P* value < 0.05 was considered statistically significant.

## Results

### Patient characteristics

According to the BSI diagnosis criteria and sample availability, 21 BSI (BSI group) and 16 non-BSI (non-BSI group) HSCT patients were observed (Fig. 1), and the characteristics were shown in Table 1. No significant differences in age or gender were found between the two groups. The primary disease of patients in BSI group was mainly acute leukemia, followed by lymphoma. In the non-BSI group, the primary diseases of patients were mainly acute leukemia and multiple myeloma. The number of allo-HSCT and auto-HSCT patients was matched in the two groups, and there was no statistical difference. Furthermore, the BSI patients’ liver function was weaker than that of the non-BSI patients in some extent, which suggested that BSI patients may have severe liver dysfunction. In addition, the pathogens in the BSI group were mainly Gram-negative bacteria, most of which were Enterobacteriaceae (38.1%, 8/21), followed by *Pseudomonas aeruginosa* (28.6%, 6/21). Gram-positive bacteria were mainly coagulase negative *staphylococcus epidermidis* (19.0%, 4/21), which is a common pathogen in HSCT patients.

**Table 1:** Patient characteristics

	BSI (n = 21)	non-BSI (n = 16)	*P* value^d^
Age at diagnosis (year)^a^	39.8 ± 15.8	41.6 ± 18.9	0.746
Gender (M/F)	M 15 F 6	M 10 F 6	0.726
Primary disease			–
AML	10	2	
ALL	2	3	
CMML	–	1	
MDS	2	2	
Lymphoma	4	–	
MM	1	6	
AA	1	1	
POEMS syndrome	1	–	
PNH	–	1	
Types of HSCT	allo-HSCT 19 auto-HSCT 2	allo-HSCT 10 auto-HSCT 6	0.055
Liver function			
ALT (U/L)^a^	70.4, 78.4	19.3, 11.5	0.001
AST (U/L)^a^	40.9, 38.7	19.0, 9.8	0.057
GGT (U/L)^a^	144.4, 151.9	42.1, 40.8	< 0.001
Total bilirubin (µmol/L)^a^	22.2, 28.6	12.2, 5.2	0.058
Direct bilirubin (µmol/L)^a^	13.24, 22.8	4.8, 2.3	0.003
BSI related pathogens^b^			–
Coagulase-Negative *Staphylococcus*^c^	4	–	
*Enterococcus faecium*	1	–	
*Streptococcus *spp.	2	–	
*Enterobacter cloacae*	2	–	
*Escherichia coli*	5	–	
*Klebsiella pneumoniae*	1	–	
*Pseudomonas aeruginosa*	6	–	
*Corynebacterium jeikeium*	1	–	

^a^ Mean ± SD; ^b^ One patient had a mixed *Staphylococcus epidermidis* and *Streptococcus oral* infection; ^c^ Eliminate possible contamination according to BSI diagnostic criteria; ^d^ Students' t-test was used for data with a homogeneous variance; otherwise, Mann–Whitney *U* test was used; Fisher's exact test was used for counts

AML: acute myeloid leukaemia; ALL: acute lymphoblastic leukaemia; CMML: chronic myelomonocytic leukemia; MDS: myelodysplastic syndromes; MM: multiple myeloma; AA: aplastic anemia; PNH: paroxysmal nocturnal hemoglobinuria

### Alterations of intestinal microbiome preceding BSI in HSCT patients

**Fig. 2:**
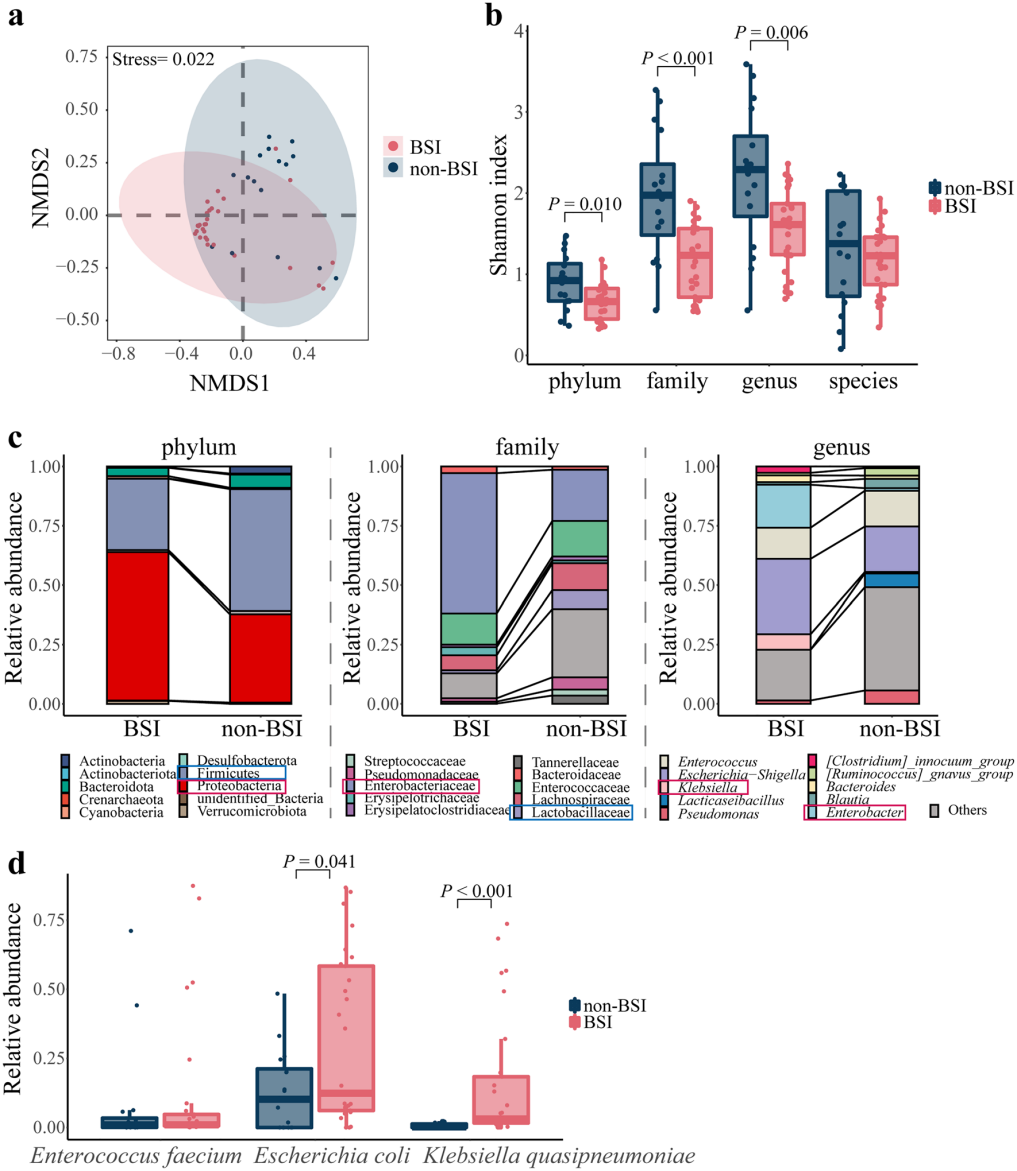
Characteristics of the intestine microbiome in BSI and non-BSI patients. **a** Analysis of non-metric multidimensional scaling (NMDS) of samples from BSI and non-BSI groups at family level. **b** Shannon diversity at the phylum, family, genus, and species levels **c** Stacked column charts of the relative abundance of the top 10 phyla, families and genera. The red and blue boxes represented a significant increase and decrease in relative abundance in the BSI group, respectively, compared with the non-BSI group **d** Boxplot of relative abundance at species level. The student’s t-test was used for data with a homogeneous variance; otherwise, the Mann–Whitney test was used. Multiple groups were compared using one-way ANOVA

To investigate intestinal microbiome alterations in HSCT patients before BSI onset, 16S rRNA gene sequencing was performed on stool specimens. The difference between the specimens in the BSI and non-BSI groups was shown using non-metric multidimensional scaling (NMDS) based on the family level (Fig. 2a). The Shannon diversity in BSI group was significantly decreased at different taxonomic levels, compared with the non-BSI group, except species level (Fig. 2b). There was no significant difference in richness between the BSI and non-BSI groups (Additional file 7: Fig. S1). The relative abundance of Firmicutes and Proteobacteria in the BSI group was 21.2% lower and 25.3% higher than that in the non-BSI group on average, respectively (Fig. 2c and Additional file 2: Table S1). The relative abundances of Lactobacillaceae (*P* = 0.031) and Tannerellaceae (*P* = 0.007) were decreased in the BSI group, while Enterobacteriaceae abundance (*P* < 0.001) was increased significantly (Fig. 2c). The relative abundances of *Enterobacter* (*P* < 0.001) and *Klebsiella* (*P* = 0.002) were increased and the *Blautia* abundance was decreased significantly (*P* = 0.027), among the top 10 genera in the BSI group (Fig. 2c). The abundance of *Enterococcus faecium* was no changed, while the abundance of *Escherichia coli* (*P* = 0.041) was increased significantly in BSI group, compared with non-BSI group (Fig. 2d). Interestingly, *K. quasipneumoniae* was significantly increased (*P* < 0.001) in the BSI group, compared with non-BSI group (Fig. 2d). These results suggested that intestinal microbiome may be altered before BSI onset, mainly manifesting reduced probiotics and increased potential pathogens.

### Microbiome features at the family level predicting BSI onset

**Fig. 3:**
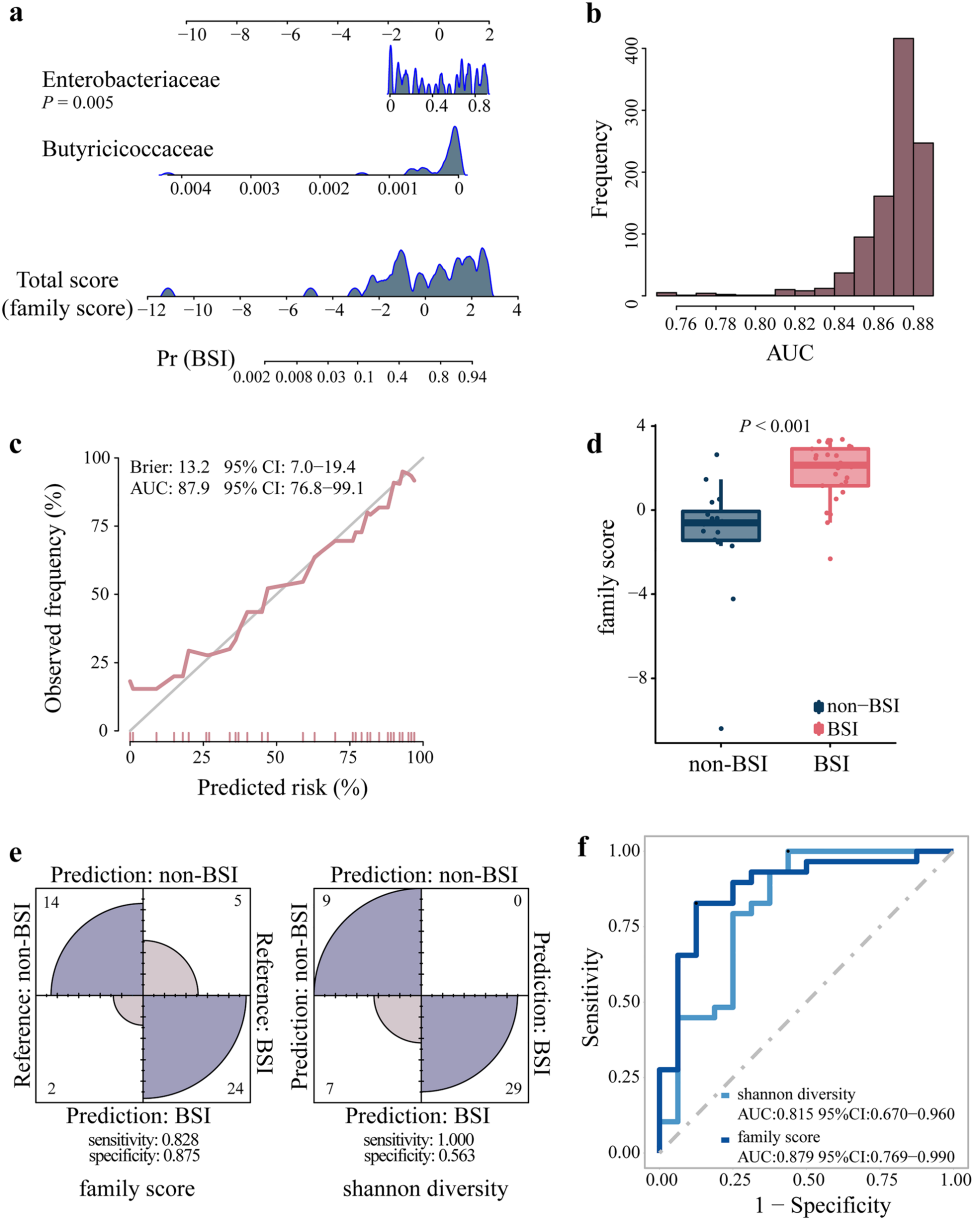
The prediction model for BSI. **a** Developed nomogram. The first and third lines represented the score and the total score scales, respectively; the last line represented the probability of BSI corresponding to the total score; and the peaks represented density. **b** Histogram of AUC frequency under 1000 sampling. **c** Calibration curves. AUC, area under the curve; CI, confidence interval. **d** The family scores’ boxplot in the BSI and non-BSI groups. **e** Prediction results’ confusion matrix.** f** Receiver operating characteristic (ROC) curves of family score and Shannon diversity at the family level. The student’s t-test was used for data with a homogeneous variance; otherwise, the Mann–Whitney test was used. Multiple groups were compared using one-way ANOVA

To observe the possible predictability of microbiome features in the early post-HSCT period, patients in the BSI and non-BSI groups were randomly divided into the training and validation sets to perform the LASSO algorithm (Additional file 7: Fig. S2). A total of 7 features were selected from 414 families using the LASSO logistic regression model (Additional file 3: Table S2). Because Enterobacteriaceae and Butyricicoccaceae could be detected in more than 90% of samples, these two features were selected for subsequent logical regression and the total score (family score) was then obtained (Fig. 3a and Additional file 3: Table S2). After validating the model using the bootstrap method, the mean AUC value was 0.869 (95% CI 0.823–0.884), and the percentage of AUC greater than 0.85 was 91.9% (919/1000) (Fig. 3b). The agreement between the prediction and observation was demonstrated by the calibration curve (Fig. 3c). The family score was significantly different between the BSI and non-BSI groups (Fig. 3d). The family score (sensitivity, 0.828; specificity, 0.875; AUC, 0.879) was showed higher predictive performance than the Shannon diversity at family level (sensitivity, 1.000; specificity, 0.563; AUC, 0.815) through confusion matrix and receiver operating characteristics (ROC) curves (Fig. 3e, f).

### Elevated serum bile acid related to alterations of intestinal microbiome

**Fig. 4:**
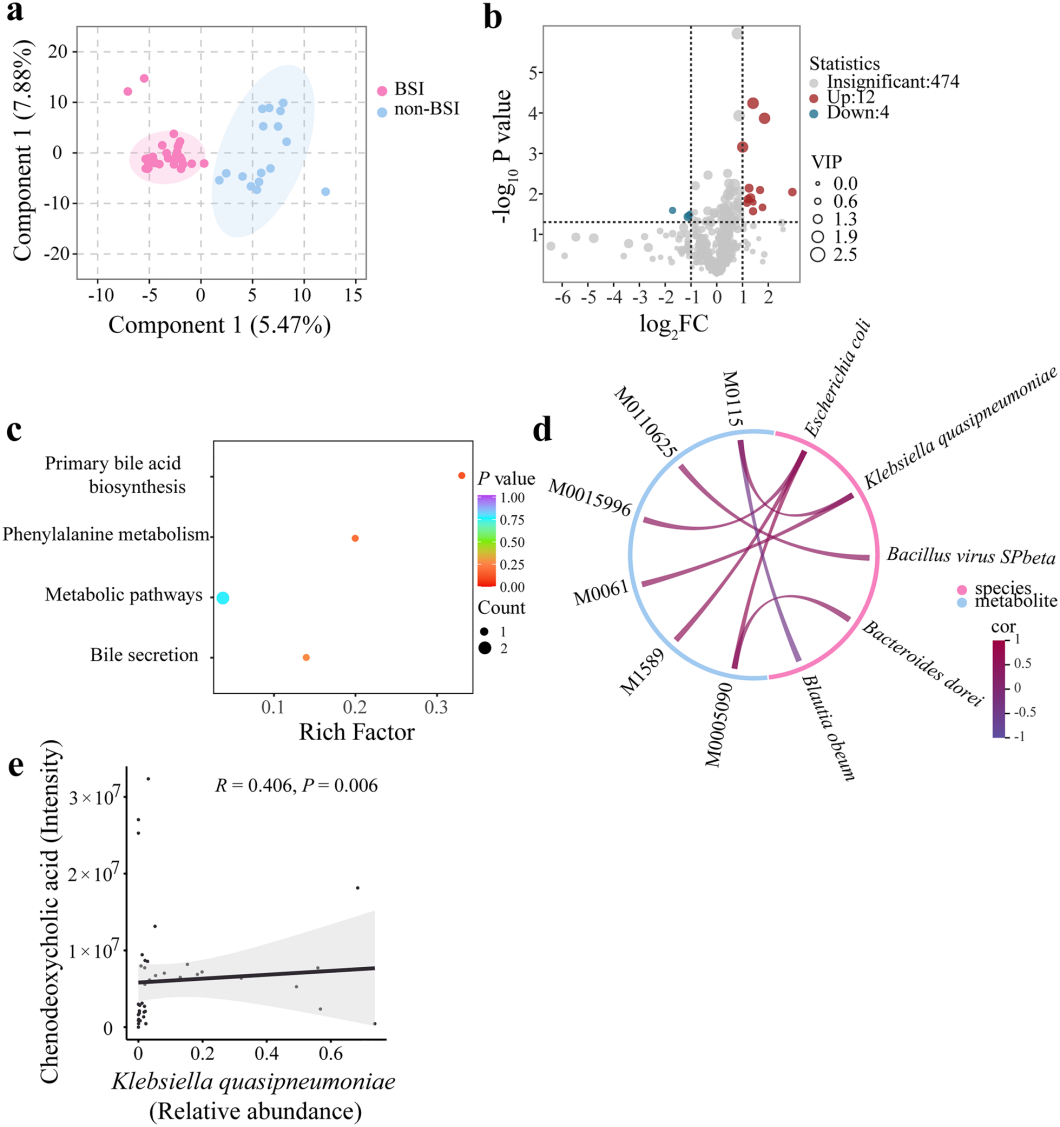
Serum metabolome and its relationship with the microbiome in HSCT patients. **a** Partial least squares-discriminant analysis combined with orthogonal signal correction (OPLS-DA) of samples from the BSI and non-BSI groups. R^2^X = 0.134; R^2^Y = 0.849; Q^2^ = 0.416. **b** Metabolites’ volcano plot. FC, fold change; VIP, variable importance projection. **c** Bubble diagram of the KEGG enrichment pathway of the differential metabolites. **d** Chord diagram of correlation between differential metabolites and top 10 species. The relationships were shown according to correlation coefficient (*R*) > 0.4 and *P* values (*P*) < 0.05. **e** Two dimensional correlation scatter plot of *Klebsiella quasipneumonae* (relative abundance) and CDCA (intensity). The student’s t-test was used for data with a homogeneous variance; otherwise, the Mann–Whitney test was used. Multiple groups were compared using one-way ANOVA

Since intestinal microbiome is closely related to host metabolism, an untargeted metabolomic assay of serum samples was performed. A remarkable difference in samples between the BSI and non-BSI groups was displayed by OPLS-DA (Fig. 4a). There were 16 differential metabolites, including 4 downregulated and 12 upregulated metabolites (Fig. 4b and Additional file 4: Table S3). KEGG enrichment analysis revealed that the differential metabolites were mainly enriched in the primary bile acid biosynthesis pathway (Fig. 4c).

Next, we attempted to investigate the effect of microbiome on host metabolism. The correlations between the intensity of differential metabolites and the relative abundance of top 10 species were analysed using spearman correlation coefficient. The results showed that there was a certain correlation between 5 species and 6 different metabolites (*R* > 0.4) (Fig. 4d). Only chenodeoxycholic acid (CDCA) (M0115) was screened among the differential primary bile acid metabolites, and its intensity was positively correlated with the relative abundance of *K. quasipneumoniae* (*R* = 0.406, *P* = 0.006) and negatively correlated with the relative abundance of *Blautia obeum* (*R* = -0.413, *P* = 0.005) (Fig. 4d, e). These findings suggested that the increased relative abundance of *K. quasipneumoniae* may lead to the elevated level of serum primary bile acid.

### Increased primary bile acid levels and its transport in mice colonized with *K. quasipneumoniae*

**Fig. 5:**
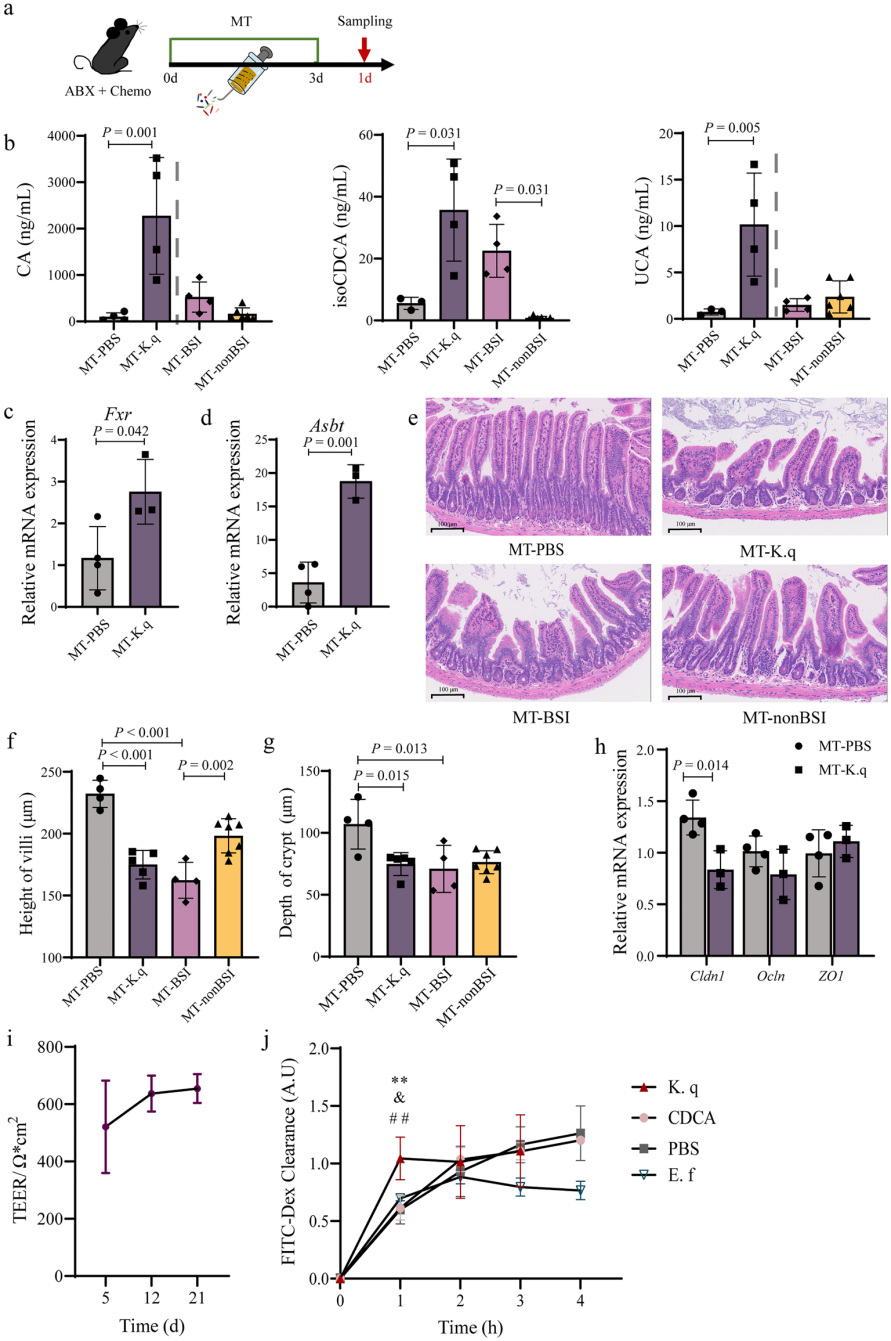
Mouse experiment with bacteria colonization. **a** Flow chart for verifying the correlation between *K. quasipneumoniae*/microbiome and bile acids in mice. ABX, MNVA in drinking water; Chemo, chemotherapy; MT, *K. quasipneumoniae* or microbiome transplantation for colonization; sampling on the first day after transplantation. **b** Concentration of serum primary bile acids in mice after transplantation. CA, cholic acid; isoCDCA, isochenodeoxycholic acid; UCA, ursocholic acid. MT-PBS, gavage with PBS; MT-K.q, gavage by *K. quasipneumoniae*; MT-BSI, gavage by microbiome from BSI patients; MT-nonBSI, gavage by microbiome from non-BSI patients.** c** and** d** Relative mRNA expression of *Fxr* and *Asbt* in MT-PBS and MT-K.q groups. **e** The pathological results of the ileum obtained by H&E staining after transplantation. **f** and **g** The villus height and crypt depth of the mice small intestine after transplantation. **h** Relative mRNA expression of *Cldn1*, *Ocln* and *ZO1* in MT-PBS and MT-K.q groups. **i** Relationship between TEER of Caco-2 monolayers and culture time. **j** FITC-dextran paracellular transport in Caco-2 monolayers. ** represented significant difference between K.q group and CDCA group (*P* = 0.005), & represented significant difference between group K.q group and E.f group (*P* = 0.014), and ## represented significant difference between K.q group and PBS group (*P* = 0.003). Bars represent the mean ± SD. The student’s t-test was used for data with a homogeneous variance; otherwise, the Mann–Whitney test was used. Multiple groups were compared using one-way ANOVA

To verify whether the relative abundance of *K. quasipneumoniae* and the level of bile acid was related, mice fed with broad-spectrum antibiotics (ABX) were treated with chemotherapy to simulate HSCT patients’ intestinal conditions and then subjected to bacterial transplantation by gavage (Additional file 7: Fig. S3). The serum samples from mice were collected on the first day after transplantation, and main bile acids were detected (Fig. 5a and Additional file 5: Table S4). Primary bile acids, including CA (*P* = 0.001), isoCDCA (*P* = 0.031), and UCA (*P* = 0.005), in mice colonized with *K. quasipneumoniae* (MT-K.q group) were significantly increased compared with the control group (MT-PBS group) (Fig. 5b). However, except isoCDCA, for the other two primary bile acids, no significant differences were observed between the mice transplanted with BSI and non-BSI patients’ microbiome (MT-BSI and MT-nonBSI groups) (Fig. 5b). It’s all known that unconjugated bile acids, like CA and CDCA, can activated bile acid signalling farnesoid X receptor (FXR), and the majority of them are then reabsorbed by apical sodium-dependent BA transporter (ASBT) into the enterocytes and transported back to the liver [33]. Our results found that the *Fxr* and *Asbt* mRNA expression in MT-K.q group were significantly higher than that in MT-PBS groups (*P* = 0.042 and *P* = 0.001, respectively), suggesting that the increased primary bile acids caused by the colonization of *K. quasipneumoniae* promoted the activation of FXR and the transport of primary bile acids (Fig. 5c, d). Furthermore, the bile acid levels (including primary and secondary) on the first (1d) and third day (3d) were also compared after bacterial transplantation (Additional file 7: Fig. S4). The primary bile acids at 3d in the MT-K.q group decreased compared with that at 1d, may suggesting that the time of colonization was insufficient (Additional file 7: Fig. S4c).

### Severe intestinal mucosa injury in mice colonized with *K. quasipneumoniae*

Next, the mice intestinal mucosa was observed because of its direct relatedness to the microbiome. The villi in MT-K.q, MT-BSI, and MT-nonBSI groups were pathologically damaged to a certain degree compared with the PBS control group at 1d, including atrophy, height decrease, and rupture (Fig. 5e). Simultaneously, the hypertrophic crypts were also observed (Fig. 5e). Compared with the MT-nonBSI group, more serious villi loss and damage were shown in the MT-BSI group (Fig. 5e). In contrast to the MT-PBS group, the small intestinal villus height and crypt depth in the MT-K.q and MT-BSI groups decreased significantly (Fig. 5f,g and Additional file 5: Table S4). These results demonstrated that the colonization of *K. quasipneumoniae* and the microbiome of BSI patients caused serious mice intestinal mucosa damage. However, there was no difference in villus height and crypt depth between MT-K.q and MT-BSI groups, indicating that *K. quasipneumoniae* may play a major role in intestinal mucosal injury (Fig. 5f, g). Then, the mRNA expression of intestinal epithelial tight junction protein relative genes were measured between MT-K.q and MT-PBS groups to analyse the integrity of intestinal mucosa, including claudins, occludin and zonula occludens (ZOs) genes. The claudin-1 gene (*Cldn1*) relative mRNA expression was significantly decreased in MT-K.q group, compared with MT-PBS group (*P* = 0.014) (Fig. 5h), showing that the colonization of *K. quasipneumoniae* destroyed the integrity of intestinal mucosa in mice by reducing the *Cldn1* expression. Moreover, compared with 1d, the 3d results showed that the small intestinal mucosa was recovered in the three transplant groups (Additional file 7: Figs. S5 and S6).

### Caco-2 cell monolayers permeabilization induced by *K. quasipneumoniae*

In order to observe the factors causing intestinal mucosal damage, Caco-2 cell monolayers was constructed and incubated with different substances to observe permeabilization. CDCA (CDCA group) and *K. quasipneumoniae* (K.q group) were selected to incubate with Caco-2 cell monolayers, respectively. Simultaneously, a co-incubation group of Caco-2 cell monolayers and *E. faecium* was set up (E.f group). In the above analysis, the relative abundance of *E. faecium* was found to have no significant difference between BSI and non-BSI patients. On the 12th to 21st days of culture, the TEER tended to be stable, proving compact monolayers successfully constructed (Fig. 5i). The FITC-dextran clearance experiment results showed that FITC clearance in K.q group at 1 h was significantly increased compared with PBS group (*P* = 0.003), CDCA group (*P* = 0.005) and E.f group (*P* = 0.014) (Fig. 5j). Subsequently, the fluorescence clearance of K.q group tended to be stable and consistent with that of the PBS group (Fig. 5j). The possible reason was that the concentration difference of fluorescein in the upper and lower compartments decreased rapidly after 1 h. The results proved that *K. quasipneumoniae*, instead of CDCA, significantly induced the permeability of intestinal cell monolayers.

## Discussion

HSCT is a common method for treating benign and malignant hematological diseases. However, infections, especially BSI, are associated with high mortality in adult HSCT patients [1]. Recently, microbiome analyses have provided a basis for better interpreting the complicated relationship between microbiome and HSCT [4]. One study found that the intestinal microbiome characteristics could predict the risk of infection in patients with acute myeloid leukaemia and HSCT [11, 12]. Alterations in microbiome-related metabolites are associated with HSCT complications [18]. Investigations on intestinal microbiome and host metabolome in patients with BSI are yet to be undertaken to discover the biological characteristics preceding BSI.

Through stool 16S rRNA gene sequencing analysis, we found that the intestinal microbiome α diversity increased gradually from the phylum to the genus level. However, the diversity decreased at the species level. This may have resulted from the 16S rRNA gene sequencing technology limitations, causing low species detection [34]. Previous studies have shown that the higher Gammaproteobacteria (including Enterobacteriaceae) abundance, the higher mortality associated with pulmonary complications after HSCT [16]. A previous study found that moderate or severe aGVHD patients prior to transplantation have high abundance of Lactobacillaceae, exhibiting high mortality [35]. However, the abundance of Lactobacillaceae was lower and Enterobacteriaceae was higher in neonates with necrotizing enterocolitis (NEC), relative to non-NEC patients [36]. The high abundance of Lactobacillaceae was also observed in patients with end-stage renal disease treated with dietary fiber, proving its benefits [37]. Another study found that Bacilli, Erysipelotrichaceae, and Enterobacteriaceae (*Klebsiella*) relative abundance were increased, and *Prevotella*, Ruminococcaceae, and *Akkermansia* relative abundance were decreased in children before HSCT compared with the healthy controls [38]. Consistent with previous studies, our results also found that Enterobacteriaceae relative abundance was increased, but Lactobacillaceae was decreased before BSI onset. Therefore, the negative effects of Enterobacteriaceae and positive effects of Lactobacillaceae may be indicated [38].

In previous studies, *Enterococcus, Streptococcus,* and various Proteobacteria were dominant during HSCT. Among them, enterococcal dominance increased vancomycin-resistant *Enterococcus* bacteremia risk by nine folds, whereas proteobacterial domination increased Gram-negative rod bacteremia risk by five folds [13, 39]. Although *Enterococcus* was not significantly different between the BSI and non-BSI groups in our study, the increased potentially harmful bacteria (especially Enterobacteriaceae reported) and decreased beneficial bacteria under serious disease conditions were proven, and its predictability for BSI was confirmed. A marked difference in *K. quasipneumoniae* between the BSI and non-BSI groups was also observed. This opportunistic pathogen species could cause gastrointestinal tract infections [40]. We also found that the colonization of *K. quasipneumoniae* can induce intestinal mucosal barrier damage in mice. Therefore, further studies are needed to explore whether the intestinal barrier damage caused by high abundance of *K. quasipneumoniae* in HSCT patients is a related to BSI occurrence. Due to the close correlation between the microbiome and host metabolism [8], serum metabolome detection and its association with the microbiome were determined in our study. Differential metabolites were mainly enriched in the primary bile acid biosynthetic pathway. Abnormal bile acid metabolism was consistent with liver injury in HSCT patients with BSI in this study. These findings suggested that HSCT patients with elevated bile acid levels may be related to subsequent BSI onset. Bile acid metabolites could modulate immune cells to regulate host immunity [39, 41]. Altered host immunity caused by bile acids might be a possible reason for BSI occurrence in HSCT patients. In our results, although primary bile acid did not injury the intestinal mucosal barrier, its impact on host immunity remains to be confirmed in the future.

Moreover, bile acid biotransformation results from the host and intestinal microbiome interaction. Primary bile acid deconjugation occurs via bile salt hydrolases (BSH), widespread in the microbiome. Firmicutes, Bacteroidetes, and Actinobacteria with BSH have been identified in metagenomic studies [42]. This seems to be a possible reason to explain why the primary bile acids in HSCT patients with BSI were elevated in our study, because the abundance of Firmicutes and Actinobacteria was decreased. Furthermore, the physiological function of the host can be altered by microbiome-related secondary bile acids [42]. LCA and DCA may harm the intestine and contribute to intestinal diseases, including membrane damage and colon cancer. However, ursodeoxycholic acid (UDCA) protects colon cells from apoptosis and oxidative damage [42–44]. Although other major secondary bile acids in our results did not show differences between the infection and non-infection groups except for 7-KDCA (Additional file 7: Fig. S4b), the secondary bile acids’ role in HSCT patient needs to be explored because of intestinal susceptibility.

The increased serum primary bile acids might be associated with increased *K. quasipneumoniae* according to the correlation analysis in this study. Therefore, *K. quasipneumoniae* and primary bile acids were believed to be related and subsequently verified in mice. Since intensive chemotherapy could destroy the microbiome composition and further affect bile acid production, a chemotherapy mouse model was constructed to simulate the intestinal conditions of HSCT patients [45]. We confirmed that the intestinal colonization of *K. quasipneumoniae* in mice increased serum primary bile acids. However, an important point that needs to be explored is whether bile acid is catabolized or anabolized by *K. quasipneumoniae*. Wei Jia et al. reviewed that alternative bile acid (BA) synthetic pathway may be manipulated by gut microbiota [46]. Therefore, it is necessary to explore the genes related to bile acid metabolism in *Klebsiella quasipneumoniae* and set up the *K. quasipneumoniae* colonization group with inhibitors to deeply analyse the specific relationship between them. Furthermore, the mice transplanted with BSI patients’ microbiome showed increased primary bile acids compared with those transplanted with non-BSI patients’ microbiome to a certain extent. This was consistent with our findings in HSCT patients. Interestingly, CDCA was elevated in BSI patients, whereas isoCDCA was increased in mice transplanted with BSI patients’ microbiome. IsoCDCA is a CDCA stereoisomer [47, 48]. Therefore, the effect of interaction between *K. quasipneumoniae* and other bacteria within the microbiome on metabolism still need to be studied and discussed further.

A limitation of this study is the small number of final cases. As it was impossible to predict which patient with HSCT would have BSI, collecting samples prospectively on a large scale was necessary. Only 25% of HSCT patients have BSI. Among these patients, those with available samples were further filtered. Although 75% of patients did not meet the BSI diagnostic criteria, most of them did not rule out the possibility of infection clinically or had no samples available. Therefore, only 21 BSI and 16 non-BSI patients were finally included in this study. In a previous prospective single-centre study, 19 eligible HSCT children were included [49]. Twenty-two out of 113 patients developed bacteraemia in an allogeneic HSCT were studied [13]. Therefore, the difficulty in collecting samples before BSI may be indirectly explained. However, the sample size must be expanded to improve relevant studies in the future.

## Conclusions

This study demonstrated that the microbial diversity and probiotics were decreasing, and potential pathogens (especially *K. quasipneumonae*) were increasing the HSCT patient before BSI onset. This study also revealed the host metabolic profile before BSI, confirmed the relationship between *K. quasipneumonae* and serum primary bile acid, and emphasized the effect of *K. quasipneumonae* on the damage of mucosal barrier. Furthermore, the microbiome features at family level were highly predictive of BSI and could be further used as potential biomarkers.

## Supplementary Information

**Additional file 1.** Supplementary Materials and Methods. Method details of human stool 16S rRNA gene sequencing and serum untargeted metabolomics analysis.

**Additional file 2:** **Table S1.** The relative abundance and microbial diversity in different taxonomy.

**Additional file 3:** **Table S2.** The results of LASSO and logisitic regression.

**Additional file 4. Table S3.** The differential metabolites of human serum.

**Additional file 5. Table S4.** The detection of serum bile acids and small intestinal in mice.

**Additional file 6. Table S5.** The list of all primers for PCR.

**Additional file 7:** Supplementary figures. Fig. S1 to Fig. S6.

## Data Availability

All data generated or analysed during this study were included in this published article and its supplementary information files. The data has been submitted to the NCBI BioProject database (PRJNA874351).

## Abbreviations

HSCT: Hematopoietic stem cell transplantation
GVHD: Graft-versus-host disease
BSI: Bloodstream infection
OTU: Operational taxonomic units
LASSO: Least absolute shrinkage and selection operator
TEER: Transepithelial electrical resistance
CDCA: Chenodeoxycholic acid
NMDS: Non-metric multidimensional scaling
ROC: Receiver operating characteristics
ABX: Broad-spectrum antibiotics
FXR: Farnesoid X receptor
ASBT: Apical sodium-dependent BA transporter
