# Supplementary material for: Klebsiella quasipneumoniae in intestine damages bile acid metabolism in hematopoietic stem cell transplantation patients with bloodstream infection
Source: J Transl Med. 2023 Mar 29;21:230. doi: 10.1186/s12967-023-04068-9 (PMC10061697; doi:10.1186/s12967-023-04068-9)
Supplement: Supplementary file 1 — Additional file 1. Supplementary Materials and Methods. Method details of human stool 16S rRNA gene sequencing and serum untargeted metabolomics analysis. [file 12967_2023_4068_MOESM1_ESM.docx]

Method details of human faeces 16S rRNA gene V3-V4 regions sequencing and serum untargeted metabolomics analyses

# Supplemental materials and methods

## Human intestinal microbiome

## *Faeces collection and DNA extraction*

All clinical residual faeces samples were collected and refrigerated at –80 ℃ within 24 hours. The genomic DNA of faeces was extracted by DNeasy PowerSoil Pro Kit (QIAGEN, Germany). Then, the purity and concentration of DNA were detected by 1% agarose gel electrophoresis. The DNA was diluted with sterile water to 1 ng/μL.

## *16S rRNA gene V3-V4 region sequencing*

The diluted genomic DNA was used as a template. 16S rRNA V3-V4 regions were amplified used specific primers with the barcode. All PCR reactions were performed with 15 μL Phusion^®^ High-Fidelity PCR Master Mix (New England Biolabs); 2 μM forward and reverse primers, and about 10 ng template DNA. The PCR products were detected by electrophoresis on 2% agarose gel and the target products were purified with Gel Extraction Kit (Qiagen, Germany). Sequencing libraries were constructed by TruSeq^®^ DNA PCR-Free Sample Preparation Kit (Illumina, USA). The library quality was assessed on the Qubit 3.0 (Thermo Scientific, USA) and Agilent Bioanalyzer 2100 system (Aglient, USA). The constructed library was sequenced on Illumina NovaSeq6000 platform and 250 bp paired-end reads were generated.

## *Sequencing data analysis*

The barcode and primer sequences were removed from the raw data. Paired-end reads were assembled by FLASH (version 1.2.7) [1]. QIIME (version 1.9.1) [2] was used to filter high-quality clean tags. The quality control process of QIIME was followed: a) Tags interception: cut raw tags from the first low-quality base site with continuous low-quality value (default quality threshold is ≤ 19) and base number reaching the set length (default length value is 3); b) Tags length filtering: the tags data set obtained by intercepting tags further filtered out tags in which the continuous high-quality base length was less than 75% of the tags length. The tags sequence was compared with the Silva Database to remove the chimera sequence. OTU clustering was performed by UPARSE software (version 7.0.1001) [3]. Sequences with > 97% similarity were assigned to the same OTUs. Representative sequence for each OTU was screened for annotation. The species annotation was performed using Silva Database based on Mothur algorithm [4]. The community composition of each sample was counted at each classification level: Kingdom, phylum, class, order, family, genus and species. Finally, the least amount of data in the sample was used as the standard for homogenization. The subsequent alpha diversity analysis and beta diversity analysis were based on the homogenized data.

## Human serum untargeted metabolomics

## *Sample preparation and extraction*

The remaining clinical serum samples were collected within 6 hours, frozen in liquid nitrogen for 15 minutes and refrigerated at -80℃. After that, the samples were thawed on ice. 300μL ice-cold methanol (containing 1ppm of 2-chlorophenylalanine) was added to 100μL serum, vortex vibrated for 2 minutes and incubated at -20℃ for 0.5h. Then, the mixture was shaken for 2 minutes and centrifuged at 12,000 rpm at 4℃ for 10 minutes. 200μL supernatant was taken out and incubated at -20℃ for 0.5h. Finally, the supernatant was centrifuged at 12,000 rpm at 4℃ for 15 minutes. The supernatant was then collected for LC-MS analysis.

## *HPLC Conditions (T3)*

All samples were acquired by the LC-MS system followed machine orders. The analytical conditions were as follows, UPLC: column, Waters ACQUITY UPLC HSS T3 C18 (1.8µm, 2.1mmх100mm); column temperature, 40℃; flow rate, 0.4mL/min; injection volume, 2μL; solvent system, water (0.1% formic acid): acetonitrile (0.1% formic acid); gradient program, 95:5 V/V at 0 min, 10:90 V/V at 11.0 min, 10:90 V/V at 12.0 min, 95:5 V/V at 12.1 min, 95:5 V/V at 14.0 min.

## *Data processing*

The original data file obtained by LC-MS analysis is transformed into mzML format by ProteoWizard software (version 3.0). Peak extraction, alignment and retention time correction were performed using the XCMS program, and the "SVR" method was applied to correct the peak area. Filter the peaks with deletion rate > 50% in each group. Then, the metabolic identification information was obtained by searching database (Pubchem database, KEGG database, the Human Metabolome database). Finally, R program is used for statistical analysis. Statistical analysis includes univariate analysis and multivariate analysis. Univariate statistical analysis included Student’s t-test and multivariate analysis of variance [5].

# References

1. Magoč T, Salzberg SL. FLASH: fast length adjustment of short reads to improve genome assemblies. Bioinformatics. 2011;27(21):2957-63; doi: 10.1093/bioinformatics/btr507.

2. Caporaso JG, Kuczynski J, Stombaugh J, Bittinger K, Bushman FD, Costello EK, et al. QIIME allows analysis of high-throughput community sequencing data. Nat Methods. 2010;7(5):335-6; doi: 10.1038/nmeth.f.303.

3. Haas BJ, Gevers D, Earl AM, Feldgarden M, Ward DV, Giannoukos G, et al. Chimeric 16S rRNA sequence formation and detection in Sanger and 454-pyrosequenced PCR amplicons. Genome Res. 2011;21(3):494-504; doi: 10.1101/gr.112730.110.

4. Quast C, Pruesse E, Yilmaz P, Gerken J, Schweer T, Yarza P, et al. The SILVA ribosomal RNA gene database project: improved data processing and web-based tools. Nucleic Acids Res. 2013;41(Database issue):D590-6; doi: 10.1093/nar/gks1219.

5. Zou W, She J, Tolstikov VV. A comprehensive workflow of mass spectrometry-based untargeted metabolomics in cancer metabolic biomarker discovery using human plasma and urine. Metabolites. 2013;3(3):787-819; doi: 10.3390/metabo3030787.
