# Supplementary material for: Klebsiella quasipneumoniae in intestine damages bile acid metabolism in hematopoietic stem cell transplantation patients with bloodstream infection
Source: J Transl Med. 2023 Mar 29;21:230. doi: 10.1186/s12967-023-04068-9 (PMC10061697; doi:10.1186/s12967-023-04068-9)
Supplement: Supplementary file 7 — Additional file 7: Supplementary figures. Fig. S1 to Fig. S6. [file 12967_2023_4068_MOESM7_ESM.docx]

## *Fig. S1 Microbial richness in HSCT patients*


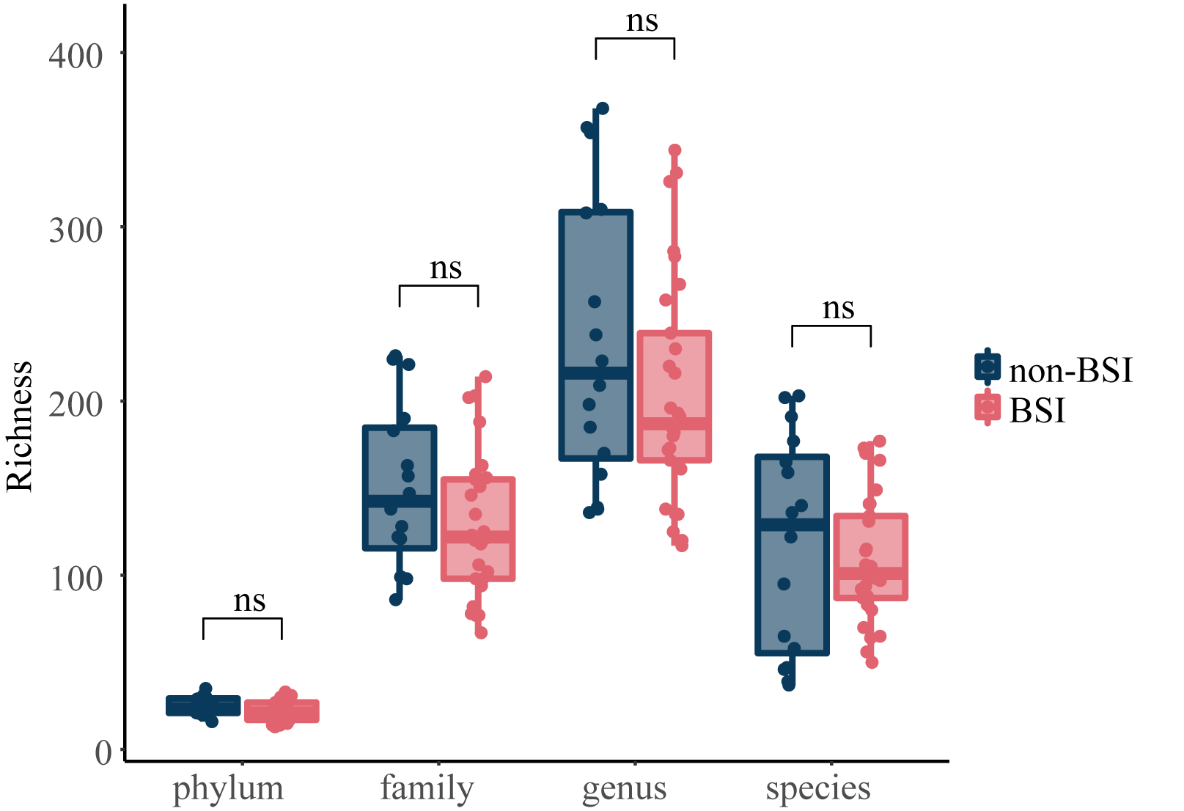


The boxplot of microbial richness (observed species) in BSI and non-BSI groups at different taxonomy.

## *Fig. S2 Construction process of infection prediction model*


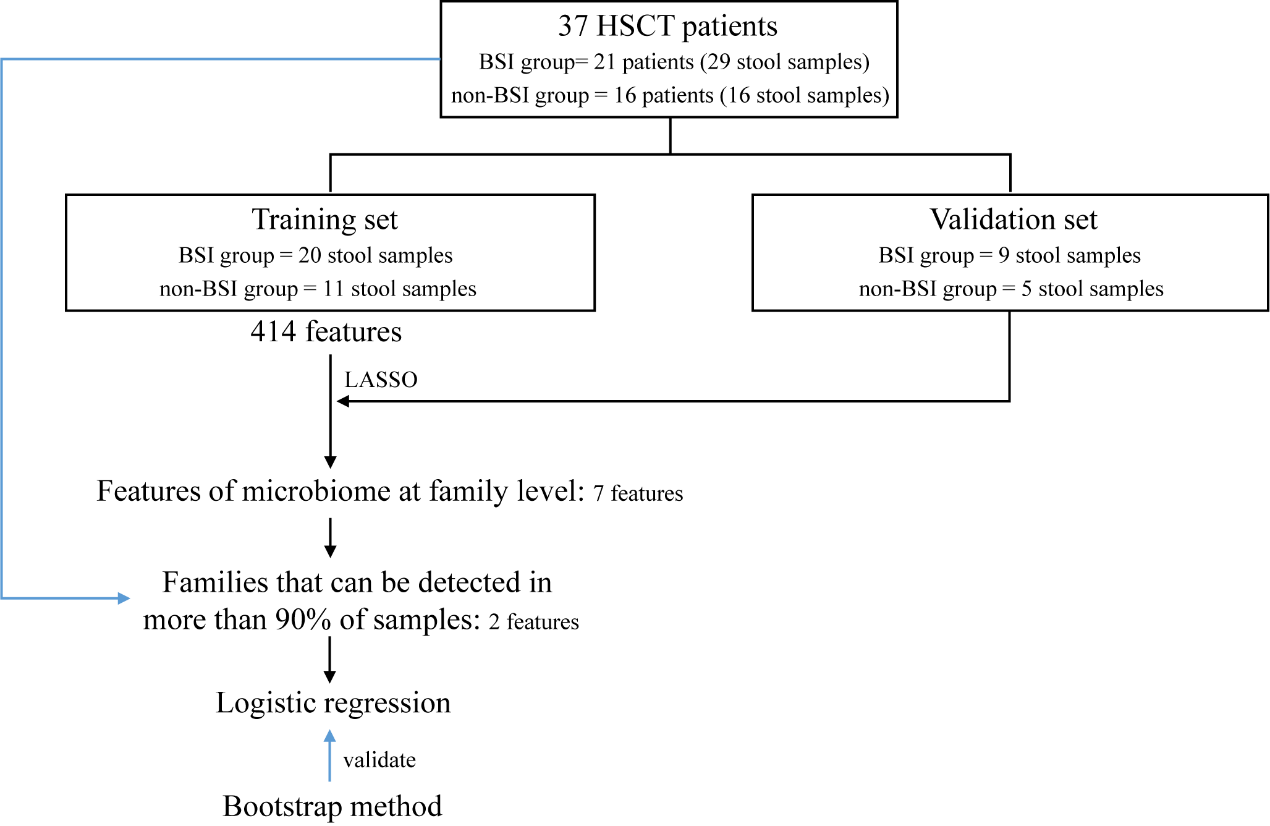


Constructing the logistic regression model. LASSO, least absolute shrinkage, and selection operator.

## *Fig. S3 Depletion of intestinal microbiome and chemotherapy in mice*


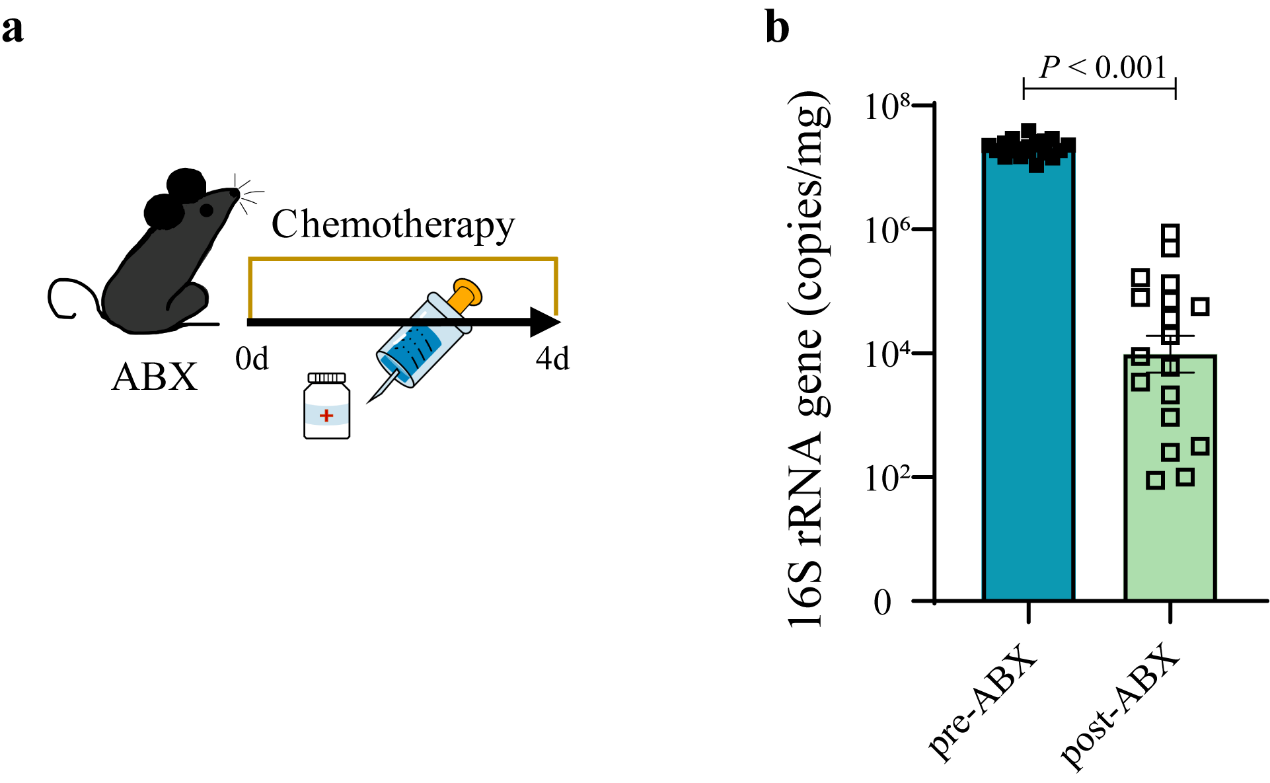


**a** Construction process of the chemotherapy mouse model. ABX, MNVA in drinking water; Chemotherapy, ABX mice injected with chemotherapeutic drugs (detailed in the “methods” section). **b** 16S rRNA gene copy numbers of mouse stool samples before and after broad-spectrum antibiotics (metronidazole, neomycin sulfate, ampicillin, and vancomycin (MNVA)). Bars represent the mean ± SD. The student’s t-test was used for data with a homogeneous variance; otherwise, the Mann-Whitney test was used. Multiple groups were compared using one-way ANOVA.

## *Fig. S4 The correlation between bile acid and Klebsiella quasipneumoniae/microbiome in mice*


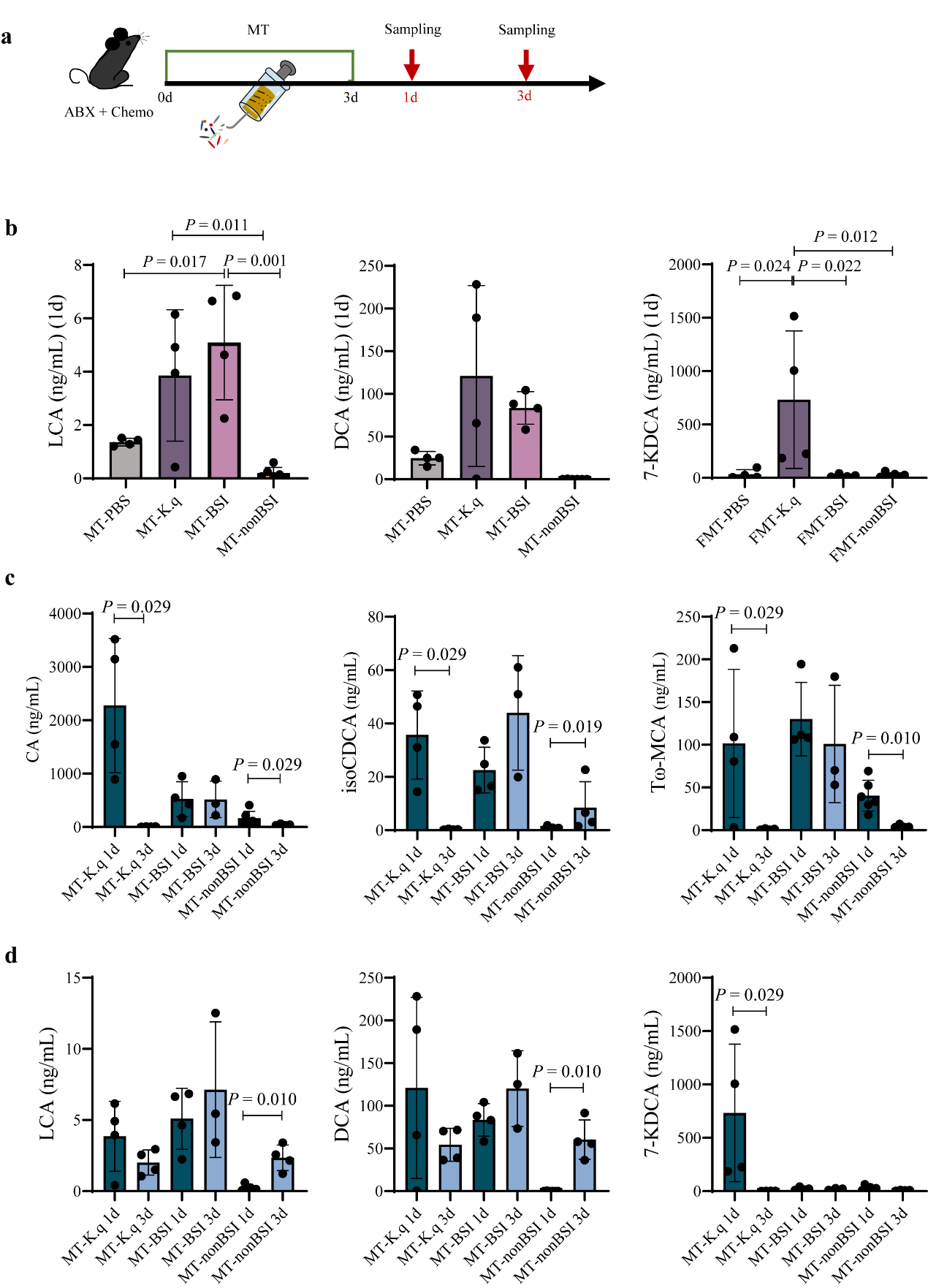


**a** Flow chart for verifying the association between *Klebsiella quasipneumoniae* and bile acids in mice. ABX, MNVA in drinking water; Chemo, chemotherapy; K.q/MT, *Klebsiella quasipneumoniae*/microbiome transplant; sampling on the first and third days after transplantation. **b** Comparing secondary bile acid production on the first day after transplantation. LCA, lithocholic acid; DCA, deoxycholic acid; 7-KDCA, 7-Ketodeoxycholic acid. MT-PBS, gavage with PBS; MT-K.q, gavage by *Klebsiella quasipneumoniae*; MT-BSI, gavage by microbiome from infected patients; MT-nonBSI, gavage by microbiome from non-infected patients. **c** Comparing primary bile acid production on the first and third days after transplantation. CA, cholic acid; isoCDCA, isochenodeoxycholic acid; UCA, ursocholic acid. **d** Comparing secondary bile acid production on the first and third days after transplant. Bars represent the mean ± SD. The student’s t-test was used for data with a homogeneous variance; otherwise, the Mann-Whitney test was used. Multiple groups were compared using one-way ANOVA.

## *Fig. S5 Mice small intestinal pathology at different sampling time points*


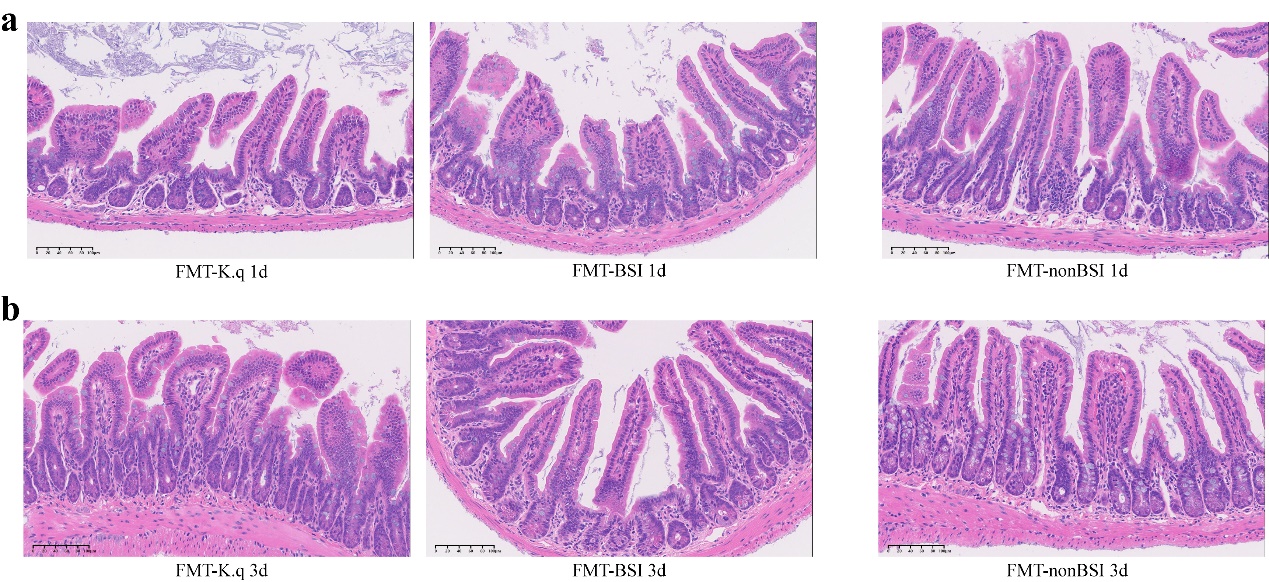


**a** Small intestinal pathological results on the first day after transplantation. **b** Small intestinal pathological results on the third day after transplantation.

## *Fig. S6 The villi height and crypts depth in mice small intestine*


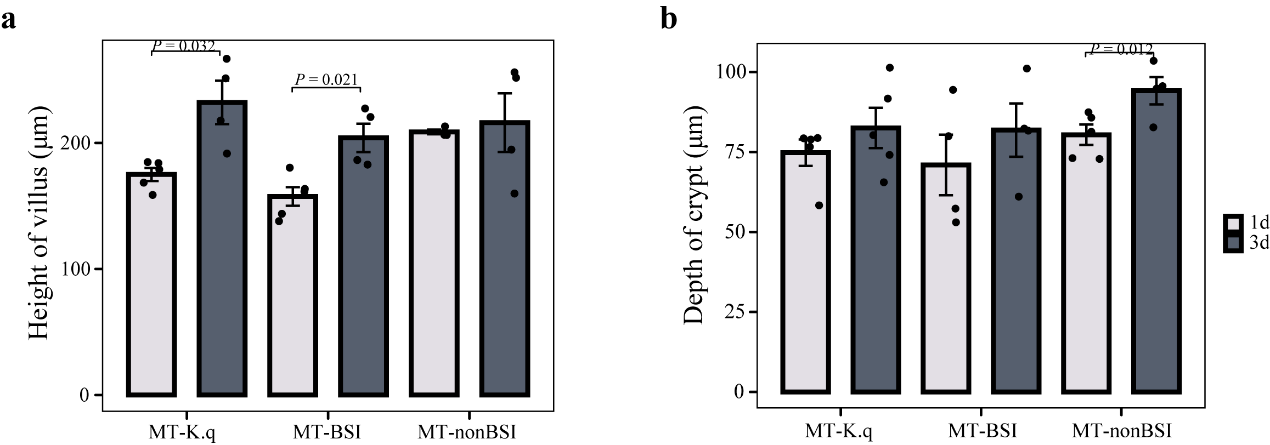


**a** and **b** Comparing the villus height and crypt depth in the small intestine at different sampling time points. Bars represent the mean ± SD. The student’s t-test was used for data with a homogeneous variance; otherwise, the Mann-Whitney test was used. Multiple groups were compared using one-way ANOVA.
